# Delayed Pulmonary Metastasis of Basal Cell Carcinoma 10 Years After Primary Excision: A Case Report and Literature Review

**DOI:** 10.1155/carm/8239242

**Published:** 2025-09-10

**Authors:** Hazhir Moradi, Negar Karavan, Forough Kalantari, Elham Kalantari

**Affiliations:** ^1^Nosocomial Infection Research Center, Isfahan University of Medical Sciences, Isfahan, Iran; ^2^Department of Internal Medicine, Isfahan University of Medical Sciences, Isfahan, Iran; ^3^Department of Nuclear Medicine, Rasoul Akram Hospital, Iran University of Medical Sciences, Tehran, Iran

**Keywords:** basal cell, carcinoma, case reports, lung neoplasms, neoplasm metastasis

## Abstract

**Background:** Basal cell carcinoma (BCC) is the most common cutaneous malignancy, characterized by slow progression and a low propensity for metastasis. Metastatic basal cell carcinoma (mBCC) occurs in fewer than 0.1% of the cases, most frequently involving the lungs, lymph nodes, or bones. Although rare, mBCC is associated with poor prognosis and presents unique diagnostic and therapeutic challenges.

**Case Presentation:** We report a 77-year-old male with a remote history of multiple head-and-neck BCCs, including aggressive histologic subtypes (basosquamous and micronodular), treated predominantly with Mohs surgery; the margin status varied across procedures (some tumor free and some positive). Ten years after the initial lesion, the patient developed progressive dyspnea and was found to have bilateral pulmonary nodules on chest CT. PET/CT demonstrated increased FDG uptake, and a CT-guided biopsy of the right lung nodule confirmed mBCC. There was no evidence of local recurrence at the original excision sites. p16/HPV studies were not performed on the prior cutaneous primaries.

**Conclusion:** This case highlights the potential for delayed pulmonary metastasis in BCC, even years after apparently curative treatment. The absence of local recurrence and the bilateral lung involvement suggest hematogenous spread. Clinicians should remain vigilant for metastatic disease in patients with a history of high-risk BCC, particularly when new pulmonary symptoms arise. Imaging and immunohistochemistry are critical for diagnosis, and early detection may improve therapeutic outcomes in this rare and aggressive manifestation. In this patient, the presence of aggressive histologic subtypes and prior positive margins likely increased metastatic risk.

**Summary**

• Pulmonary metastasis from BCC can present a decade after apparently definitive local treatment and without local recurrence.
• Aggressive histologic subtypes (e.g., basosquamous and micronodular) and prior positive margins may increase the metastatic risk.
• Sustained long-term vigilance with prompt imaging and IHC is crucial when new pulmonary symptoms arise in patients with prior high-risk BCC.

## 1. Introduction

Basal cell carcinoma (BCC) is the most common cutaneous malignancy worldwide, accounting for nearly 80% of all nonmelanoma skin cancers. It typically follows an indolent clinical course with rare potential for metastasis, which is estimated to occur in fewer than 0.1% of the cases [1, 2]. The low metastatic propensity has often led to BCC being regarded as a locally destructive yet fundamentally benign neoplasm. However, when metastasis does occur, it is associated with aggressive clinical behavior and poor prognosis [3].

Metastatic basal cell carcinoma (mBCC) most frequently involves distant sites such as the lungs, bones, and regional lymph nodes [4, 5]. Risk factors contributing to this rare transformation include neglect of primary lesions, recurrent tumors, perineural or vascular invasion, immunosuppression, and specific histologic subtypes like morphea form or basosquamous variants [3, 6]. In particular, lesions arising in the head and neck region have been implicated in a disproportionate number of metastatic cases, possibly due to enhanced vascular and lymphatic networks.

Despite decades of clinical observation, the pathophysiological mechanisms underpinning BCC metastasis remain poorly understood. Emerging data suggest a central role for aberrant Hedgehog (HH) signaling, particularly involving PTCH1 and SMO gene mutations, which drive unchecked cellular proliferation and, in rare instances, promote invasive phenotypes [7]. Recent advances in systemic therapy—namely, HH pathway inhibitors and immune checkpoint blockade—have opened new avenues for treatment, though their efficacy in metastatic disease remains limited and under ongoing investigation [8, 9].

Here, we present a rare case of mBCC with bilateral pulmonary involvement occurring 10 years after excision of primary lesions, in the absence of local recurrence. This report underscores the necessity of long-term vigilance even in ostensibly low-risk BCCs and aims to contextualize this case within the broader clinical and molecular landscape of metastatic BCC.

## 2. Case History/Examination

Over the past decade, the patient—a 77-year-old man—had developed multiple cutaneous malignancies involving the head and neck region, predominantly BCC with documented subtypes across different excisions, including basosquamous carcinoma, micronodular BCC, and nodular BCC. Margin status varied: some excisions achieved tumor-free margins, whereas others reported positive deep or lateral margins (Table 1) [10]. Surgical management was undertaken on multiple occasions, including Mohs micrographic excisions and rotational flap reconstruction. Histopathologic evaluation revealed a mixture of outcomes, with some lesions achieving tumor-free margins and others showing residual tumor at the deep or lateral margins. A summary of the patient's prior dermatologic surgeries, histologic diagnoses, and anatomical sites is provided in Table 1. Immunohistochemical assessment for p16 or HPV DNA testing was not performed on the prior cutaneous primaries. Histomorphology in the reviewed specimens favored BCC subtypes without features typical of HPV-related basaloid squamous cell carcinoma (BSCC) [11].

Most recently, the patient was referred to the pulmonary medicine clinic with a 2-month history of gradually worsening exertional dyspnea. The symptom developed insidiously and progressed to the point of interfering with his ability to perform daily activities. He denied cough, hemoptysis, chest pain, fever, night sweats, or weight loss.

His medical history was, otherwise, notable for long-term occupational exposure to ultraviolet radiation due to outdoor farming. He had no history of systemic immunosuppression, smoking, or radiotherapy. A family history of lung cancer was reported in his brother, who died at age 68.

On physical examination, the patient was in no acute distress. Vital signs were within normal limits, including an oxygen saturation of 96% on ambient air. Pulmonary auscultation was clear bilaterally, and no adventitious sounds were noted. There were no signs of peripheral edema or lymphadenopathy. Cardiovascular, abdominal, and neurologic examinations were unremarkable.

**Figure 1:**
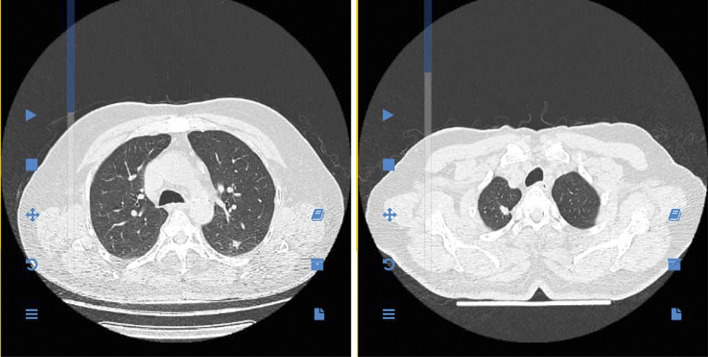
Axial chest CT showing bilateral pulmonary nodules.

Given the patient's oncologic background and recent respiratory symptoms, a high-resolution multidetector computed tomography (HR-MDCT) scan of the chest was performed. This revealed two discrete, well-defined pulmonary nodules: a 10-mm lesion in the apical segment of the right upper lobe and an 8-mm nodule in the superior segment of the left lower lobe. No mediastinal lymphadenopathy, pleural effusion, or parenchymal scarring was present (Figure 1). These findings were deemed indeterminate but suspicious for malignancy given the clinical context.

A subsequent 18F-fluorodeoxyglucose positron emission tomography/computed tomography (FDG-PET/CT) scan showed intense FDG uptake in the right upper lobe lesion (SUVmax 7.3) and moderate uptake in the left lower lobe lesion (SUVmax 2.0). Two additional subcentimeter nodules were identified but were non-FDG-avid and considered clinically insignificant.

**Figure 2:**
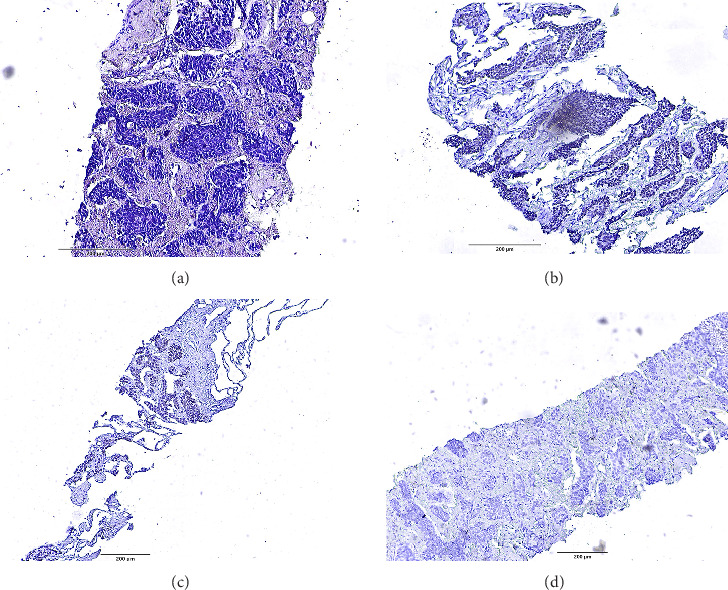
Representative microscopic images of the lesion with various staining techniques. (a) Hematoxylin and eosin (H&E) staining showing the general histoarchitecture of the lesion. (b) Immunohistochemical staining for p63 showing strong nuclear positivity in tumoral cells. (c) Duplicate view of p63 staining at a higher magnification, highlighting the distribution pattern of p63-positive cells. (d) EMA (Epithelial Membrane Antigen) staining demonstrating cytoplasmic and/or membranous positivity in the glandular components.

A CT-guided core needle biopsy was performed on the right upper lobe nodule. Histopathologic examination revealed nests of basaloid cells with peripheral palisading and mitotic figures. Immunohistochemical staining was positive for p63 and Ber-EP4 and negative for TTF-1 and CK7, confirming the diagnosis of mBCC. The morphology was consistent with the patient's previously excised cutaneous tumors (Figure 2).

### 2.1. Differential Diagnosis

1. Primary lung carcinoma
2. Metastatic squamous cell carcinoma
3. Metastasis from an unknown primary
4. mBCC (confirmed via histopathology and IHC).

### 2.2. Outcome and Follow-Up

In the absence of local recurrence at the prior surgical sites, the bilateral pulmonary nodules were interpreted as true distant metastases rather than synchronous primaries or direct extension. The patient was diagnosed with mBCC with pulmonary metastasis and was subsequently referred to a multidisciplinary tumor board for comprehensive treatment planning.

## 3. Discussion and Literature Review

### 3.1. Epidemiology and Risk Factors

BCC remains the most common cutaneous malignancy worldwide, yet metastatic transformation is exceedingly uncommon, occurring in < 0.1% of all BCC cases [12]. Contemporary population data from Australia and Western Europe confirm that the age-standardized incidence of metastatic or locally advanced BCC (la/mBCC) has remained static (≈ 1-2 per million/year) despite a steady rise in overall BCC diagnoses [12]. Established risk factors for dissemination include the following:

• Tumor related: diameter > 5 cm (so-called ∗ giant ∗ BCC), aggressive histotypes (morpheaform, infiltrative, micronodular, and basosquamous), perineural/vascular invasion, and deep (beyond subcutis) extension [10].
• Host related: immunosuppression, prior radiotherapy, male sex, and germ-line PTCH1 mutations (Gorlin–Goltz syndrome) [10].

Lesions situated in the head-and-neck region account for ∼65% of the metastatic cases, plausibly because of dense lymphovascular networks facilitating hematogenous spread [5]. Notably, our patient harbored aggressive subtypes (basosquamous and micronodular) and had variable margin status across prior surgeries—both recognized risk enhancers for dissemination [10].

#### 3.1.1. Molecular Pathogenesis

Carcinogenesis in BCC is driven by constitutive activation of the HH signaling cascade—classically through PTCH1 loss-of-function or activating SMO mutations [13]. Whole-exome sequencing of metastatic deposits has demonstrated the following:

• Secondary SMO resistance mutations (e.g., D473Y and G497W) that abrogate binding of HH inhibitors (HHIs) [13].
• Frequent coalterations in the Hippo–YAP/TAZ and PI3K/AKT pathways, providing permissive signals for invasion and epithelial–mesenchymal transition [14].
• Very high tumor-mutational burden (median ≈ 90 mut/Mb) and occasional PD-L1 amplification, furnishing a biologic rationale for response to immune-checkpoint blockade [7].

These data collectively suggest that metastatic competency in BCC arises from multistep genetic evolution beyond solitary HH dysregulation.

#### 3.1.2. Clinical Patterns and Latency

Pulmonary parenchyma is the dominant metastatic site (≈55%), followed by lymph nodes and bone [5]. Latency between primary treatment and first distant manifestation remains strikingly variable (months ⟶ decades), reflecting long-lived tumor dormancy.  Table 2 summarizes the salient recent case reports.

Differential diagnosis included HPV-related BSCC, which has risen in head-and-neck sites. In our case, the pulmonary biopsy showed a BCC immunophenotype (p63/BER-EP4 positive and TTF-1/CK7 negative). p16/HPV testing was not performed on the cutaneous primaries; however, the available histomorphology across prior excisions favored BCC subtypes without the overt squamous differentiation typical of HPV-related BSCC [11].

#### 3.1.3. Systemic Therapy Landscape

First-generation HHIs (vismodegib and sonidegib) achieve overall response rates of 30%–58% but median durability is < 1 year; acquired resistance is usually mediated by SMO missense variants [18]. Immune checkpoint inhibitors (ICIs) have reshaped the therapeutic algorithm: cemiplimab obtained FDA approval in 2021 for la/mBCC after HHI failure, with updated data showing a 31% objective response and 2-year overall survival of 64% [17]. Combination strategies (sequential or concurrent HHI + ICI) and neoadjuvant HHI to downstage unresectable disease are under active investigation (e.g. NCT05533788) [19].

#### 3.1.4. Guidelines and Future Directions

The 2023 EDF/EADO/EORTC consensus guideline now defines mBCC as any histologically proven BCC with distant organ involvement irrespective of nodal status and recommends PET/CT staging for high-risk primaries [10]. Future translational work is focusing on YAP inhibitors, interferon-stimulated gene upregulation, and bespoke neoantigen vaccines for ultramutated tumors.

Although BCC is widely recognized for its indolent behavior and low metastatic potential, cases of mBCC challenge this perception and demand closer clinical scrutiny. The present case highlights a rare but clinically significant instance of pulmonary metastasis arising a decade after excision of the primary lesion, without any evidence of local recurrence. This temporal latency and atypical progression underscore the unpredictable nature of mBCC and emphasize the need for individualized long-term follow-up strategies.

Pulmonary metastasis is the most frequently reported site in mBCC, consistent with patterns observed in other cases summarized in Table 1 [1, 5]. The bilateral nature of the nodules in this patient, combined with the absence of other systemic findings or local recurrence, points to hematogenous dissemination from the primary cutaneous site. Importantly, this dissemination occurred despite histologically confirmed clear margins during prior Mohs micrographic surgery—a reminder that even definitive excision does not completely eliminate metastatic risk in high-risk tumors [2, 4].

The risk factors in this case—head and neck lesion location, multiple prior BCCs, advanced age, and prolonged sun exposure—are consistent with established predictors of metastatic behavior [3]. The long-standing nature of his initial tumor may have provided an opportunity for early vascular invasion and dormant micro metastatic seeding. This aligns with theories of “tumor dormancy,” in which disseminated cells remain clinically silent for years before reactivating due to changes in host immunity or microenvironment [9, 20].

Imaging played a critical role in this case. The initial HRCT findings were nonspecific but suggestive, prompting PET/CT evaluation, which confirmed FDG-avid nodules. FDG-PET, while not specific for BCC, remains useful for staging in atypical or advanced cases [21]. Biopsy and immunohistochemistry (IHC)—particularly p63 and Ber-EP4 positivity—were instrumental in differentiating mBCC from other primary lung neoplasms or metastases of unknown origin [11].

Therapeutic options for mBCC remain limited and controversial. Surgery is rarely feasible in disseminated disease, and conventional chemotherapy has shown minimal efficacy. HH pathway inhibitors such as vismodegib and sonidegib have demonstrated response rates of 30%–60% in advanced BCCs, though resistance and relapse are common [18, 22]. Recently, ICI, particularly PD-1 blockers like cemiplimab, have emerged as promising alternatives with early evidence of durable responses in mBCC [23]. However, most data derive from phase II trials, and access remains limited outside of academic centers.

From a prognostic standpoint, median survival in mBCC remains poor—ranging from 8 to 24 months depending on metastatic burden and response to systemic therapy [24]. While our patient's disease was confined to the lungs at diagnosis, the bilateral and FDG-avid nature of the lesions suggests a substantial tumor burden and portends a guarded outlook.

This case, like many in the literature, is limited by its retrospective nature and lack of histological confirmation of both nodules. However, the imaging findings, clinical course, and IHC results provide strong circumstantial evidence supporting the diagnosis. Clinicians should be aware that even seemingly low-risk BCCs can metastasize, and persistent or unexplained pulmonary symptoms in patients with prior BCC warrant thorough evaluation.

## 4. Conclusion

This case adds to the growing body of literature documenting the metastatic potential of BCC and illustrates the importance of long-term vigilance. It supports the utility of advanced imaging, histopathology, and multidisciplinary evaluation in the diagnosis and management of mBCC. As systemic therapies evolve, early identification of metastatic disease may allow for improved clinical outcomes in this historically refractory malignancy. This case reinforces that even “clinically cured” BCC may harbor long-term metastatic potential when aggressive histopathologic variants are present.

## Figures and Tables

**Table 1:** Summary of prior surgical excision of skin lesions.

No.	Histopathologic diagnosis	Anatomic site	Surgical technique	Margin status
1	Basosquamous carcinoma	Juxtauricular region	Rotational flap excision	Not reported
2	Basal cell carcinoma (BCC)	Left cheek	Mohs micrographic surgery	Tumor-free margins
3	Basal cell carcinoma (BCC)	Scalp	Mohs micrographic surgery	Positive margins
4	Nodular BCC	Periorbital region	Mohs micrographic surgery	Positive margins
5	Micronodular BCC	Forehead	Mohs micrographic surgery	Tumor-free margins

**Table 2:** Contemporary pulmonary metastatic BCC case reports (2016–2024).

Study (year)	Primary site	Metastatic site(s)	Latency to metastasis	Key features
Ikeda et al., 2016 [7]	Unknown (multiple primaries)	Lung, liver	5 years	Pd-l1 amplification; exceptional response to nivolumab
Li et al., 2019 [15]	Left shoulder collision tumor	Lung, axillary node	0.5 year	Basal-squamous collision; vismodegib ⟶ limited benefit
Kim et al., 2022 [16]	Left cheek	Lung, trachea, bronchus	9 years (1st), 6 years (2nd)	Recurrent endobronchial spread; multiple metastasectomies
Fordham et al., 2023 [17]	Axillary fold	Lungs	7 months	Sonidegib resistance; deep response to cemiplimab
Present case, 2025	Forehead & cheek	Lungs	10 years	Bilateral fdg-avid nodules; p63/ber-ep4 positive
