# Correction to “Delayed Pulmonary Metastasis of Basal Cell Carcinoma 10 Years After Primary Excision: A Case Report and Literature Review”

**DOI:** 10.1155/carm/9830530

**Published:** 2026-03-03

H. Moradi, N. Karavan, F. Kalantari, and E. Kalantari, “Delayed Pulmonary Metastasis of Basal Cell Carcinoma 10 Years After Primary Excision: A Case Report and Literature Review” *Case Reports in Medicine*, 2025 (2025). https://doi.org/10.1155/carm/8239242.

In the article titled “Delayed Pulmonary Metastasis of Basal Cell Carcinoma 10 Years After Primary Excision: A Case Report and Literature Review,” a figure was omitted in error. The omitted figure depicts the PET scan image of the patient, which provides further clarification of the article’s findings. This error was introduced by the authors during manuscript preparation and the figure is shown below and listed as Supplementary Figure 1:


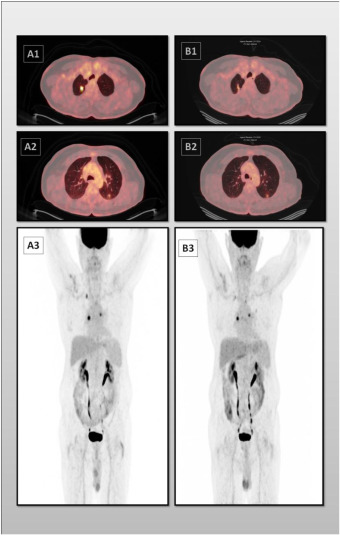


[Supplementary Figure 1: Axial fused PET‐CT and whole‐body maximum intensity projection (MIP) images before (A1–A3) and after (B1–B3) radiofrequency ablation (RFA).(A1, A2) Axial fused PET‐CT images demonstrate an 11 × 11 mm FDG‐avid nodule in the apicoposterior segment of the right upper lobe (SUVmax = 7.3, blood pool = 3.9) and a 9‐mm FDG‐avid nodule in the apical segment of the left lower lobe (SUVmax = 2.1, blood pool = 2.2). (A3) Whole‐body MIP image shows bilateral pulmonary FDG uptake without additional abnormal findings. (B1–B3) Post‐treatment PET‐CT images following RFA demonstrate a marked reduction in metabolic activity of the treated lesion, with no evidence of new abnormal FDG uptake elsewhere in the body.]

We apologize for this error.

